# Strengthening of Mg Alloy with Multiple RE Elements with Ag and Zn Doping via Heat Treatment

**DOI:** 10.3390/ma16114155

**Published:** 2023-06-02

**Authors:** Rui Fan, Lei Wang, Sicong Zhao, Liping Wang, Erjun Guo

**Affiliations:** 1 Key Laboratory of Advanced Manufacturing and Intelligent Technology (Ministry of Education), School of Material Science and Chemical Engineering, Harbin University of Science and Technology, Harbin 150000, China; fanrui_2325@163.com (R.F.); zscwr@163.com (S.Z.); lp_wang2003@126.com (L.W.); 2 School of Mechanical and Electrical Engineering, Qiqihar University, Qiqihar 161000, China; 3 The Engineering Technology Research Center for Precision Manufacturing Equipment and Industrial Perception of Heilongjiang Provincae, Qiqihar 161000, China; 4 The Collaborative Innovation Center for Intelligent Manufacturing Equipment Industrialization, Qiqihar 161000, China

**Keywords:** Mg alloy, multiple rare earth elements alloying, Ag and Zn doping, microstructure, tensile properties

## Abstract

Strengthening Mg alloys with rare earth elements has been a research focus for several decades. To minimize the usage of rare earth elements while enhancing mechanical properties, we adopted the strategy of alloying with multiple rare earth elements, namely Gd, Y, Nd, and Sm. Additionally, to promote the precipitation of basal precipitate, Ag and Zn doping was also induced. Thus, we designed a new cast Mg-2Gd-2Y-2Nd-2Sm-1Ag-1Zn-0.5Zr (wt.%) alloy. The microstructure of the alloy and its relevance to mechanical properties in various heat treatment conditions were investigated. After undergoing a heat treatment process, the alloy demonstrated exceptional mechanical properties, with a yield strength of 228 MPa and an ultimate tensile strength of 330 MPa achieved through peak-aging for 72 h at 200 °C. The excellent tensile properties are due to the synergistic effect of basal γ″ precipitate and prismatic β′ precipitate. In its as-cast state, its primary mode of fracture is inter-granular, whereas in the solid-solution and peak-aging conditions, the predominant mode of fracture is a mixture of trans-granular and inter-granular fractures.

## 1. Introduction

Mg alloys with low weight have been prosperous for several decades in the industries of aerospace, aircraft, automotives, etc., due to their high specific strength and fuel efficiency [1,2,3]. Nevertheless, the limited strength of the material restricts its extensive usage in high-end applications. To address this issue, considerable attention has been directed towards enhancing the mechanical properties of Mg alloys [4,5,6,7,8]. Research has shown that incorporating rare earth (RE) elements through alloying is a viable approach to strengthen Mg alloys [9,10,11].

Mg–RE alloys have high mechanical properties, which are mainly achieved via heat treatment (solution + aging, T6) to obtain a large number of nano-scale prismatic precipitates (β″ or β′) [12,13]. Among them, Mg-Gd alloy has become a research focus because the precipitation strengthening effect of it is the most significant [14]. However, only when the Gd content in a binary Mg-Gd alloy is beyond 15% can it show a good precipitation strengthening effect, and the addition of excessive Gd leads to increased cost and density. Most recently, research has indicated that the Mg-Gd-RE ternary alloy obtained by replacing part of the Gd with an appropriate amount of RE would exhibit a better age-hardening response. For example, precipitation strengthening has enabled Mg-8Gd-3Nd [15] and Mg-7Gd-5Y [16] alloys to achieve ultimate tensile strengths (UTS) of up to 271 MPa and 258 MPa, respectively, following peak-aging. Additionally, by alloying with double RE elements, Y and Nd, properly instead of Gd, an ultra-high UTS of 293 MPa was achieved in a Mg-8Gd-2Nd-1Y alloy after peak-aging treatment [17]. The decrease in the total amount of RE is due to the fact that the addition of multiple RE elements can reduce their solubility in Mg, thereby forming a supersaturated solid solution with less RE content [7,10]. Inspired by this idea, we designed a multiple RE-strengthened, Mg-2Gd-2Y-2Nd-2Sm-based Mg–RE alloy to obtain prismatic precipitates with less RE (8 wt.%) and thereby reduce the density as well as the cost of the alloy.

In addition, the basal precipitate can be formed by adding non-RE alloying elements such as Ag and Zn to a Mg alloy [18,19,20]. For instance, Ag doping in Mg-Gd and Mg-Gd-Y alloys can form fine and dense flake basal precipitates in an α-Mg matrix, and the formation of these basal precipitates does not significantly affect the formation of prismatic precipitates [21,22,23]. Moreover, it was reported that Ag doping would strengthen Mg-Nd alloys significantly [24,25,26] and promote the age-hardening response of Mg-Gd [27,28,29] and Mg-Y-Zn alloys [30]. If both prismatic and basal precipitate can be induced into the matrix to form a closed structure so that the strengthening effects of each precipitate are superimposed, it will be an effective strengthening strategy. Additionally, Zr is usually used as a refiner for cast Mg alloys.

Taking all of these factors into account, in this investigation, in order to develop high performance Mg alloys, we designed a Mg-2Gd-2Y-2Nd-2Sm-1Ag-1Zn-0.5Zr alloy. By alloying with multiple RE elements, the precipitation strengthening effect of the alloy with a lower total RE was ensured. Though alloying with Zn and Ag, basal precipitate was induced into the matrix, and it cooperated with the prismatic precipitate to obtain a closed structure and enhance the precipitation strengthening effect. Furthermore, we investigated how varying heat treatment conditions affected the microstructure and mechanical properties of the alloy. This research lays a foundation for the advancement of high-performance Mg alloys. The designed alloy is expected to be used in aerospace, automobile manufacturing, and other fields where low weight and high strength are urgently needed.

## 2. Materials and Methods

The raw materials used include pure Mg, Zn, and Ag (purity > 99.9% at.%), Mg-30Gd, Mg-30Y, Mg-20Sm, and Mg-30Nd master alloys. A steel crucible was used during melting in a well-type resistance furnace (SG2, Harbin Chengyan Heat treatment equipment Manufacturing Co., Ltd., Harbin, China), and the melting process was protected by adding RJ2 covering agent. The melt was stirred, left to rest, slagged, and poured into a preheated steel mold at 200 °C (mold cavity volume 90 × 10 × 60 mm^3^) and raised to a melting temperature of 750 °C to obtain as-cast samples. The as-cast samples were solute-treated at 510 °C for 12 h and then aged at 200 °C for different periods of time ranging from 2 h to 132 h.

Phase composition was analyzed using an X-ray diffractometer (XRD) (X’Pert PRO, PANalytical B.V., Almelo, The Netherlands) with Cu Kα. The scanning rate was 8°/min, and the scanning range was 10°~90°. The microstructure was characterized using an optical microscope (OM) (XD30M, Beijing Instant Hengye Technology Co., Ltd., Beijing, China), a scanning electron microscope (SEM) (Apreo C, Thermo Fisher Scientific Inc., St. Bend, OR, USA) equipped with an energy-dispersive X-ray spectrometer (EDS), and a transmission electron microscope (TEM) (JEM2100, JEOL, Beijing, China). The samples for OM and SEM characterizations were first ground with silicon carbide papers until smooth and polished with diamond polishing paste. The polished samples were then etched with picric acid solution (35 mL ethanol, 10 mL deionized water, 4 mL acetic acid, and 5 g picric acid). The TEM sample was prepared by grinding it to a thickness of ~50 μm, blanked into a small wafer with a diameter of 3 mm, and then ion-milled with 5 kV of ion gun energy and at a 4° milling angle.

The room temperature tensile tests were conducted using a universal testing machine (MTS E44, MTS Systems Co., Eden Prairie, MN, USA). The tensile rate was 1 mm/min. The dog-bone-shaped tensile specimens had a gauge dimension of 15 mm (l) ×3 mm (w) × 2 mm (t). The tensile fracture morphology was characterized via SEM. To guarantee the precision of the tensile test, five samples were assessed, and the final result was determined by calculating the average value.

The average grain size of the alloy was measured using the Nano Measurer 1.2.5 software according to the line interception method described in the ASTM E112-96 standards [31,32]. Using Photoshop 2018, the volume fractions of each phase were determined based on the measured area (in pixels) of the SEM images.

## 3. Results and Analysis

### 3.1. Microstructure of As-Cast Alloy

**Figure 1:**
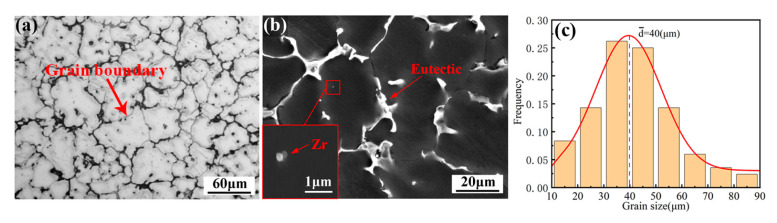
Microstructure of as-cast alloy: (**a**) OM and (**b**) SEM; (**c**) grain size distribution.

**Figure 2:**
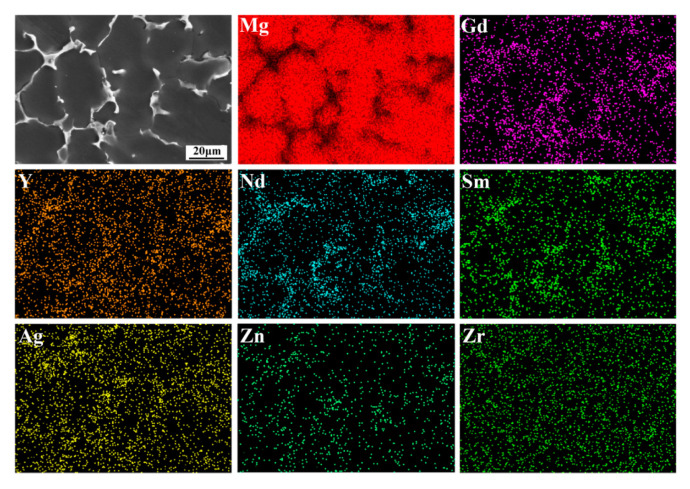
EDS mapping of as-cast alloy.

**Figure 3:**
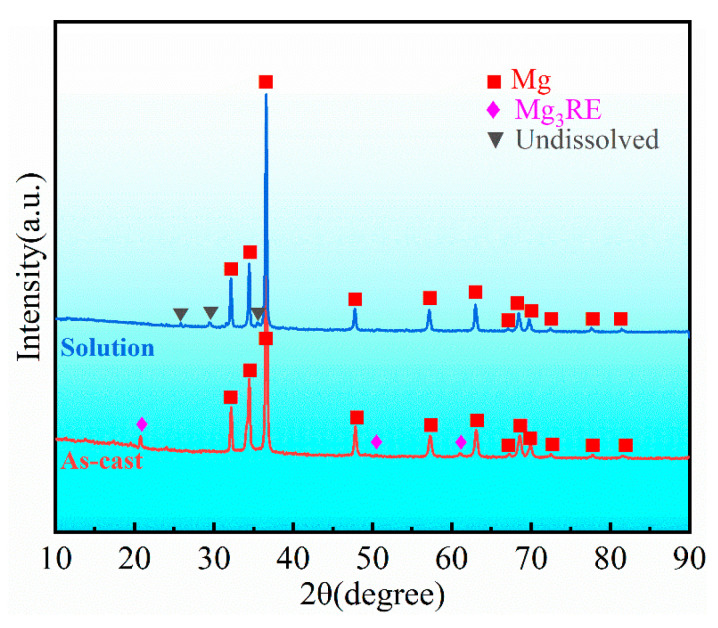
XRD patterns of alloy in as-cast and solid-solution conditions.

The microstructure of the as-cast alloy sample is illustrated in Figure 1, exhibiting a relatively homogeneous structure composed mainly of an α-Mg matrix and a reticular eutectic phase. The average grain size of the alloy was 40 ± 3 µm, its distribution is shown in Figure 1c, and the content of the reticular eutectic phase was about 6.8%, which was mainly distributed along the grain boundary. Additionally, granular phases with a diameter of about 0.5 µm could be found in the grains. The EDS mapping of the as-cast alloy is presented in Figure 2. As can be seen from the figure, the eutectic compound was enriched with RE elements and also contained some Ag and Zn elements. The EDS results for each phase are presented in Table 1, which indicates that the granular phase corresponded to Zr nuclear, which served as heterogeneous nuclei for the α-Mg matrix during solidification. The XRD patterns presented in Figure 3 offer insights into the phase composition of the as-cast alloy. Except for the Mg matrix, only peaks corresponding to the Mg_3_RE phase could be detected, which indicates that the reticular phase was a Mg_3_RE-type phase. The Zr nuclear usually cannot be detected via XRD due to its low volume fraction.

### 3.2. Solution Treatment

**Figure 4:**
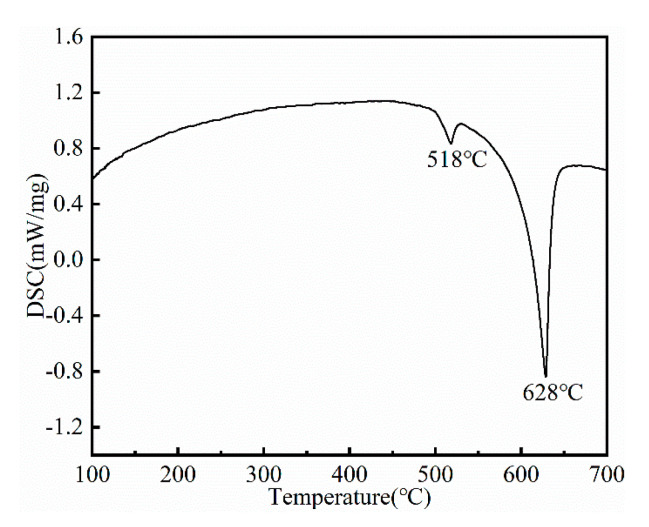
DSC curve of as-cast alloy.

**Figure 5:**
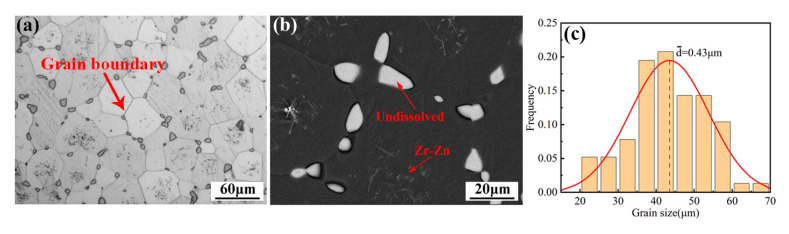
Microstructure of the alloy after treatment with solution: (**a**) OM; (**b**) SEM; (**c**) grain size distribution.

**Figure 6:**
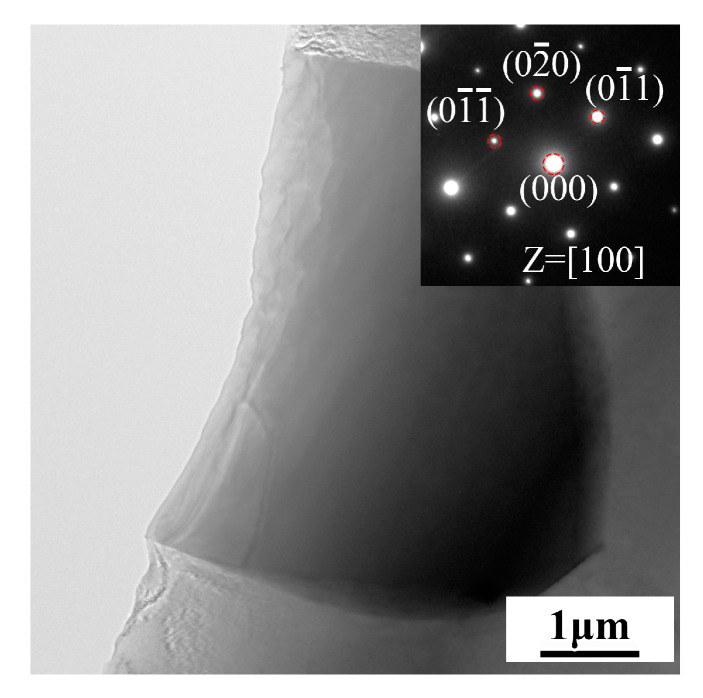
The bright field TEM image of solution-treated alloy and SAED. Inset corresponds to undissolved phase.

In order to specify the temperature for solution treatment, the DSC test was conducted, and the resulting curve is shown in Figure 4. There are two endothermic peaks in the DSC curve, which are located at 518 °C and 628 °C, respectively. The peak at 518 °C corresponds to the temperature at which the reticular phase dissolved. Therefore, the temperature for solution treatment was determined to be 510 °C to prevent overheating. Figure 5 illustrates the microstructure of the alloy after undergoing solution treatment. Compared with the as-cast alloy, the average grain size of the solution-treated alloy increased slightly to 43 ± 3 µm. Additionally, a small amount of the second phase was still retained at the grain boundary, and the fraction was about 2.1%. As presented in Table 2, the EDS results indicate that the undissolved phase mainly contained Mg, RE (Gd, Y, Nd, and Sm), Ag, and Zn elements, which is similar to the reticular phase in the as-cast alloy. However, as shown in the XRD pattern (Figure 2), the diffraction peaks corresponding to undissolved phases are clearly different from the Mg_3_RE phase. By comparison, the corresponding phase of the extra diffraction peak may be a Mg_41_Nd_5_-type phase. In addition, after solution treatment, a small amount of aggregated acicular phase precipitated in the grains. The EDS analysis (Table 2) shows that this phase was enriched in Zn and Zr elements, indicating the presence of a Zn-Zr phase, which is common in solid-solution Mg–RE alloys with high Zn content [33]. The undissolved phase was characterized via TEM, as illustrated in Figure 6, and the corresponding selected area election diffraction (SAED) pattern taken along [100] its axis is presented in the inset. The results revealed that the undissolved phase was the Mg_41_Nd_5_-type phase, which was characterized by a tetragonal structure with lattice parameters of a = 15.01 Å and c = 10.21 Å. The reason for the phase evolution is that the solubility of Nd element in Mg is the lowest [34]; in addition, the radii of Nd atoms are larger than those of other RE elements; thus, the diffusion rate in the solid-solution process was lower than that of other RE elements [35]. Therefore, during the solid-solution process, the volume of Nd dissolved into the matrix was the least among the RE elements, and thus, it undissolved compounds dominated by Nd were formed.

### 3.3. Age Hardening Behavior and Precipitate

**Figure 7:**
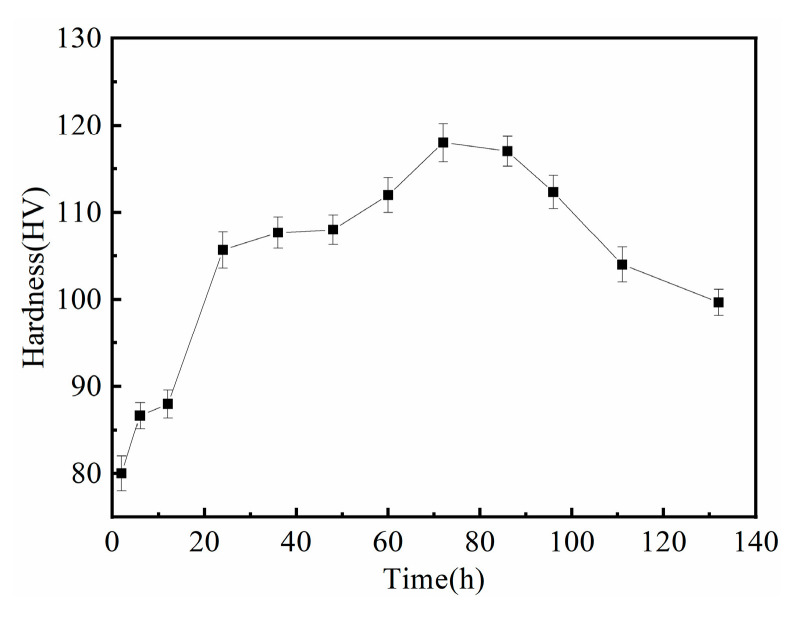
Age hardening curve at 200 °C.

Considering the precipitation strengthening effect and the time to reach peak aging, we chose 200 °C as the aging temperature. Figure 7 exhibits the age-hardening curve of the alloy at a temperature of 200 °C. It can be seen from the figure that the alloy had an exceptional age-hardening effect. The hardness of the alloy after solid-solution treatment was 80 HV; its peak hardness, 118 HV, was achieved after aging for 72 h, and the hardness decreased slowly after peak aging. The results show that the Mg–RE alloy with multiple RE elements in this study had a good age-hardening effect. The peak hardness increased by ~50% of that of the solution-treated alloy. In addition, the incorporation of Ag and Zn has been found to improve aging kinetics and peak hardness significantly in comparison to other Mg–RE-based alloys that do not contain Ag and Zn [36,37].

**Figure 8:**
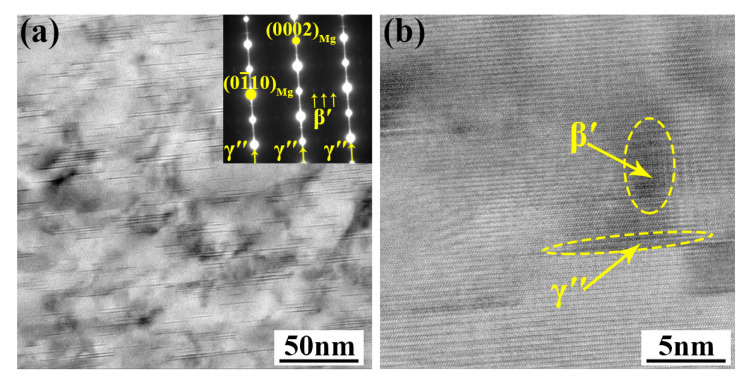
(**a**) The bright field TEM image of the peak-aged alloy taken along the [11$\bar{2}$0]_Mg_ axis (inset is SAED pattern of precipitates); (**b**) HRTEM image of alloy under peak-aging condition.

To investigate precipitation behavior during aging, the microstructure of the alloy in peak-aging condition was characterized using TEM. Figure 8 shows the TEM images taken along the [11$\bar{2}$0]_Mg_ zone axis to characterize the precipitate in the peak-aged alloy. As shown in Figure 8a, a large amount of fine precipitates was precipitated along the (0002)_Mg_ plane in the matrix. The length of the precipitated phase was 20~40 nm, and the thickness was less than 1 nm. In the corresponding SAED inset in Figure 8a, diffusion streaks parallel to the [0001]_Mg_ direction at the (0000)_Mg_ and {0110}_Mg_ positions (marked by arrows) can be detected, which are a typical feature of basal γ″ precipitates [23,38]. Additionally, extra weak diffraction spots at 1/4($0\bar{1}10$)_Mg_, 2/4($0\bar{1}10$)_Mg_, and 3/4($0\bar{1}10$)_Mg_ were also detectable in the SAED pattern. These weak diffraction spots are a typical feature of the formation of the β′ phase [39]. The β′ precipitate and α-Mg matrix had an orientation relationship of (001)_β′_//(0001)_Mg_, [100]_β′_//[11$\bar{2}$0]_Mg_ [40]. The HRTEM image, as shown in Figure 8b, further confirms the existence of β′ and γ″ precipitates in the alloy after peak-aging. In addition, the precipitates were perpendicular with each other to form a closed structure, as expected.

### 3.4. Relevance between Tensile Properties and Mechanical Properties

**Figure 9:**
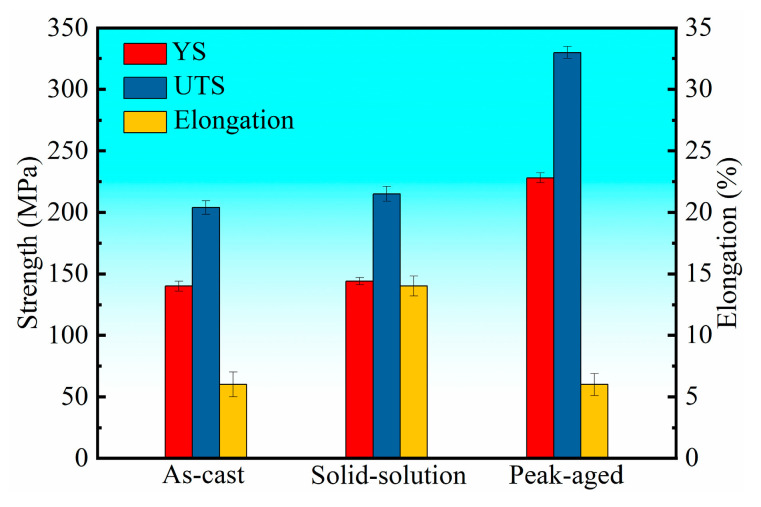
Room temperature tensile properties of the alloy in different heat treatment conditions.

Figure 9 displays the tensile properties of alloy in as-cast, solution, and peak-aging conditions. UTS increased by 5.4%, reaching 215 ± 6 MPa, and elongation significantly increased by 133.3%, reaching 14.0 ± 1.1%, as the as-cast alloy was solution-treated. Additionally, in the solution-state alloy, a slight increase in the yield strength (YS) of the alloy occurred, increasing to 144 ± 3 MPa. After aging at 200 °C for 72 h, the strength of the peak-aged alloy increased significantly as elongation decreased. The UTS, YS, and fracture elongation were 330 ± 5 MPa, 228 ± 5 MPa, and 6.0 ± 0.9%, respectively. The room temperature tensile properties of casting Mg-Gd-based alloys are listed in Table 3 for comparison. It can be seen that the alloy had good strength while maintaining a certain elongation. The mechanical properties of the alloy are closely related to its microstructure. For the as-cast alloy, there was a large amount of reticular eutectic Mg_3_RE phase, which is hard, brittle, and not coherent with the α-Mg matrix [41]. It is easy for stress to concentrate in the coarse reticular phase during deformation and reduce the alloy’s mechanical properties; thus, the as-cast alloy showed reduced strength and poor plasticity. After solid-solution treatment, the coarse reticular phase at the grain boundary dissolved into the matrix. Although there was a small amount of undissolved second phase, the fraction was obviously reduced, and the morphology changed to a more granular shape compared to the as-cast alloy (Figure 5b). As a result, after solution treatment, the stress concentration was mitigated, leading to a notable improvement in the alloy’s mechanical properties, particularly in the plasticity of the alloy. The significant improvement in the mechanical properties of the alloy in peak-aging conditions can be attributed to the synergistic effect of prismatic β′ precipitates and basal γ″ precipitates. Through adopting the strategy of alloying with multiple RE elements instead of Gd alone, a better strengthening effect was achieved with lower amounts of RE elements, generating a large amount of prismatic β′ precipitates. Additionally, the introduction of Ag and Zn promoted the formation of basal γ″ precipitates. The precipitates perpendicular to each other formed a closed structure, which has an excellent blocking effect on dislocation movement [23,42] and thereby strengthened the mechanical properties of the alloy significantly. The plasticity remained unchanged after peak aging compared to the as-cast alloy.

### 3.5. Fracture Mechanism

**Figure 10:**
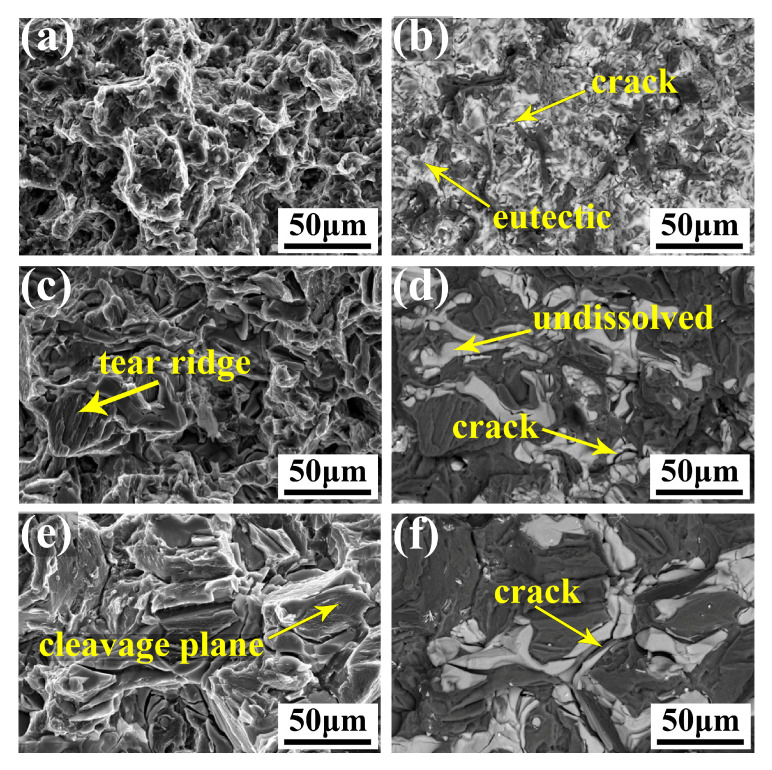
The fracture surface morphologies of the alloy: (**a**,**b**): as-cast; (**c**,**d**): solid solution; (**e**,**f**): peak-aging; (**a**,**c**,**e**): second electron images; (**b**,**d**,**f**): corresponding SEM-BSE images.

Figure 10 depicts the fracture surface morphologies of the alloy in its as-cast, solid-solution, and peak-aging conditions, which were analyzed to investigate the influence of the precipitates on the alloy’s fracture behavior. In the fracture process, crack initiation is mainly related to the type, shape, distribution, and fractions of the precipitates. In the as-cast alloy, the eutectic phase was reticular and large in size, which easily produces stress concentration; thus, in the process of plastic deformation, a crack easily sprouted in the coarse eutectic phase (Figure 10b) and propagated at the grain boundary, resulting in larger tearing edges (Figure 10a). Observably, the inter-granular fracture is the predominant fracture mode in the as-cast condition. For the solution-treated alloy, the eutectic compound was dissolved into the matrix. Although undissolved phase still existed, the volume fraction of the second phase decreased, and the sharp corner of the phase at the grain boundary was spheroidized, which relieved the stress concentration caused by the irregular and coarse eutectic phase. The presence of tearing edges within the grain, as depicted in Figure 10c, suggests that the solution-treated alloy exhibited higher plasticity compared to the as-cast alloy. Moreover, the secondary crack can be observed in the undissolved phase (Figure 10d), and the fracture mode transformed to a mixed fracture of inter-granular and trans-granular. In the peak-aged alloy, prismatic β′ precipitate and basal γ″ precipitate were uniformly dispersed in the grains, which hinders plastic deformation in the grains, thereby reducing the initiation of cracks in the grains and making the cleavage surface in the grains smooth. The crack mainly appeared on the grain boundary, and with the propagation of the crack, the intermetallic phase broke at the grain boundary, and the fracture mode was a trans-granular and inter-granular mixed fracture.

## 4. Conclusions

In this investigation, we designed a new Mg-2Gd-2Y-2Nd-2Sm-1Ag-1Zn-0.5Zr alloy, and a detailed characterization and analysis were carried out to examine its microstructure and its correlation with its mechanical properties. The following conclusions can be drawn from the experimental results:

(1) The as-cast alloy possesses an average grain size of 40 ± 3 µm, predominantly comprising an α-Mg matrix and a reticular Mg_3_RE phase enriched with RE, Ag, and Zn. After solid-solution treatment, the grain size increases slightly to 43 ± 3 µm, and the undissolved second phase of the alloy mainly transforms to a granular Mg_41_Nd_5_-type phase (a = 15.01 Å and c = 10.21 Å).
(2) The strategy of adopting multiple RE elements to alloy a Mg alloy instead of a single RE element, as well as introducing Ag and Zn doping, is effective to obtain remarkable aging hardening effects. The hardness increased from 80 HV in solid-solution conditions to 118 HV after peak-aging for 72 h at 200 °C.
(3) After peak-aging for 72 h at 200 °C, the alloy exhibits outstanding tensile properties. The UTS, YS, and fracture elongation of the tested alloy were 330 ± 5 MPa, 228 ± 5 MPa, and 6.0 ± 0.9%, respectively. These excellent tensile properties are due to the synergistic effect of basal γ″ precipitate and prismatic β′ precipitate.
(4) The alloy exhibits varying fracture behavior depending on different heat treatment conditions. In its as-cast state, the primary mode of fracture is inter-granular, whereas in the solid-solution and peak-aging conditions, the predominant mode of fracture is a mixture of trans-granular and inter-granular fractures.

## Figures and Tables

**Table 1:** EDS analysis results of second phase in as-cast alloy (at.%).

Phase	Mg	Gd	Y	Sm	Nd	Zn	Ag	Zr
Reticular	91.86	1.15	1.69	0.79	1.94	1.41	1.16	--
Granular	90.61	--	--	--	--	--	--	9.39

**Table 2:** EDS analysis results of second phase in the solid-solution alloy (at.%).

Phase	Mg	Gd	Y	Sm	Nd	Zn	Ag	Zr
Undissolved	86.53	1.17	0.98	2.21	5.73	1.85	1.55	--
Zr-Zn	53.5	--	--	--	--	18.5	--	28.0

**Table 3:** Room temperature tensile properties of Mg-Gd-based casting alloys.

Alloy	UTS (MPa)	YS (MPa)	Elongation (%)
Mg-17Gd-0.5Zr [42]	322	278	3.5
Mg-10Gd-5Y-0.4Zr [43]	302	289	2.9
Mg-11Gd-2Nd-0.5Zr [44]	345	231	2.3
Mg-14Gd-5Sm-0.3Zr [45]	286	262	0.5
Mg-8Gd-1Dy-0.4Zr [46]	355	261	3.8
Mg-8.1Gd-2.81Ho-0.38Zr [47]	279	175	4.7
Mg-8Gd-2Nd-1Y-0.6Zr [17]	293	221	4.6
This work	330	228	6.0

## Data Availability

The data presented in this study are available on request from the corresponding author.
